# Acceptance of Tele-Technology-Based Mind-Body Classes for Individuals With Mild Cognitive Impairment

**DOI:** 10.1093/geroni/igab046.435

**Published:** 2021-12-17

**Authors:** Kara Cohen, Patricia Griffiths, Tracy Mitzner

**Affiliations:** 1 Georgia Institute of Technology, Atlanta, Georgia, United States; 2 GA Tech, Atlanta, Georgia, United States; 3 Georgia Institute of technology, Atlanta, Georgia, United States

## Abstract

Individuals with Mild Cognitive Impairment (MCI) face many challenges, including cognitive declines and reduced independence which are associated with poor health outcomes. Although there is no cure for MCI, mind-body exercise classes may improve cognitive function and reduce risk of falls (Wayne, Yeh, & Mehta, 2018). However, such classes are often not accessible for individuals with MCI due to lack of transportation, fear of being stigmatized, or inability to find instructors who have experience working with individuals with MCI (Hobson & Middleton, 2008; Rimmer, 2005). Tele-technology, such as video-conferencing software, has the potential to remove barriers to participation by allowing individuals to attend classes from home. The goal of this study was to assess the feasibility of using tele-technology to deliver mind-body classes to individuals with MCI. We evaluated technology acceptance and usability for OneClick.chat, a web-based video-conferencing platform designed for older adults. Stakeholders (4 subject matter experts, 2 individuals with MCI, and 2 care partners) participated in a user study that included questionnaires and a short interview. The technology acceptance data indicate that OneClick.chat was perceived as easy to use. Some individuals expressed privacy and security concerns which could be addressed with additional education and support. These findings have implications for interface design, education, and training for deployment of tele-technology delivered mind-body classes for those with MCI.

